# Auto-Antibodies and Their Association with Clinical Findings in Women Diagnosed with Microscopic Colitis

**DOI:** 10.1371/journal.pone.0066088

**Published:** 2013-06-11

**Authors:** Bodil Roth, Rita J. Gustafsson, Bodil Ohlsson

**Affiliations:** Department of Clinical Sciences, Department of Gastroenterology, Skåne University Hospital, Malmö, Lund University, Lund, Sweden; Sanjay Gandhi Medical Institute, India

## Abstract

**Background:**

Microscopic colitis (MC) is a disease manifested by diarrhoea and is divided into collagenous and lymphocytic colitis. The aetiology is unknown, but auto-immunity is suggested. Auto-antibodies have been only rarely examined in this entity. The aim of the study was to examine the prevalence of auto-antibodies, and to examine associations between the presence of antibodies and clinical findings.

**Methods and Findings:**

Women with MC verified by biopsy and younger than 73 years, at any Department of Gastroenterology, in the district of Skåne, between 2002 and 2010 were invited to participate in this study. The patients were asked to complete both a questionnaire describing their medical history and the Gastrointestinal Symptom Rating Scale (GSRS). Blood samples were collected. Anti-nuclear antibodies (ANA), anti-neutrophil cytoplasmic antibodies (ANCA), anti-Saccharomyces cerevisiae antibodies (ASCA), and antibodies against glutamic acid decarboxylase (anti-GAD), islet antigens-like insulin 2 (anti-IA2), thyroid peroxidase (anti-TPO), and thyrotropin receptor (TRAK) were analysed. Of 240 women identified, 133 were finally included in the study, median age 63 (59–67) years. Apart from the MC diagnosis, 52% also suffered from irritable bowel syndrome, 31% from hypertension and 31% from allergy. The prevalence of ANA (14%), ASCA IgG (13%), and anti-TPO antibodies (14%) for these patients was slightly higher than for the general population, and were found together with other concomitant diseases. Patients had more of all gastrointestinal symptoms compared with norm values, irrespective of antibody expression.

**Conclusions:**

Women with MC have a slightly increased prevalence of some auto-antibodies. These antibodies are not associated with symptoms, but are expressed in patients with concomitant diseases, obscuring the pathophysiology and clinical picture of MC.

## Introduction

Microscopic colitis (MC) is a disease with watery diarrhoea without endoscopically inflamed colonic mucosa, and is divided into two different entities, collagenous colitis (CC) and lymphocytic colitis (LC). The diagnostic criterion for LC is >20 intraepithelial lymphocytes/100 enterocytes, reactive surface epithelium and mixed inflammatory infiltrate in the lamina propria. When also the subepithelial collagen band is >10 µm thick, the diagnosis CC is set [1]. The aetiology is unknown, but an auto-immune process has been proposed due to the responsiveness to corticosteroids, and a high frequency of a HLA haplotype and TNF alpha gene polymorphism (-308) associated with susceptibility to several auto-immune diseases [2]. Furthermore, other auto-immune diseases are found in 40% of these patients, thyroid diseases, rheumatologic diseases, diabetes mellitus, and coeliac disease being the most common [3], [4]. Microscopic colitis may be a different entity in younger than in older patients, where drug treatment may be a considerable aetiology of the colitis, and therefore should in these cases rather be classified as a secondary disease [1].

A high prevalence of several auto-antibodies is found in patients with auto-immune diseases [5], [6]. Antibodies against anti-neutrophil cytoplasmic antibodies (ANCA) are found in 40%–70% of patients with ulcerative colitis, and anti-Saccharomyces cerevisiae antibodies (ASCA) are found in 30%–70% of patients with Crohńs disease [7], [8]. Although MC is categorised as an inflammatory bowel disease (IBD) of auto-immune origin [9], only a few, small studies have been performed to examine the prevalence of auto-antibodies in this entity. No increased levels of rheumatoid factor or antibodies against thyroglobulin, microsomal antigen, endomysium and transglutaminase were found [3], [4]. In CC, a tendency to increased anti-nuclear antibodies (ANA) was seen in one study, whereas increased levels of ANA, ANCA, and ASCA were seen in others [3], [4], [7]. One confounding factor may be that the level of auto-antibodies correlates with disease activity, and high values may only be detected in the active disease [10].

Type 1 diabetes mellitus is an auto-immune disease associated with MC [3], [4]. Auto-antibodies that develop against islet antigens-like insulin 2 (IA2) and glutamic acid decarboxylase (GAD) are the markers of the disease, and are present in 70%–80% of cases [11], [12]. In the majority of diabetes cases, immune reaction against islet antigens and consequent formation of auto-antibodies begins long before the disease is diagnosed clinically [13]. The prevalence of these antibodies in MC has been only little examined.

The aim of this study was to further examine the prevalence of auto-antibodies in a larger cohort of MC patients, and if present, to examine the association between the presence of antibodies to concomitant diseases and clinical findings.

## Methods

### Ethics Statement

The study protocol was approved by the Ethics Committee of Lund University, and all participants gave their written, informed consent when taking part in the study (LU 2009/565 and 2011/209).

### Patients

Women who had been treated for MC at any outpatient clinic of the Departments of Gastroenterology, throughout the district of Skåne, between 2002 and 2010, were identified by a search for the ICD-10 classification for CC and LC (K52.8) in outpatient records and the local register at the Department of Pathology, Skåne University Hospital, Malmö. About one-third of the total number of the cases identified was not invited due to their being over 73 years of age, because they had many other concomitant diseases and drug therapies, which could obscure the picture with several cases of secondary MC [1]. Only the 240 patients (median 63 years, range 22–73 years) who had the diagnoses verified by colonic biopsy, and were 73 years of age or younger, were invited to participate in the present study. Altogether, 159 (median 63 years, range 22–73 years) of the 240 patients invited (66%) were recruited to the study and 134 patients (56%) also agreed to provide blood samples. One patient was excluded due to another IBD diagnosis a few weeks after the inclusion, leaving 158 (66%) and 133 patients (55%), respectively, to be included in the final calculations and laboratory analyses. These patients represent the majority of female cases of diagnosed MC in the southernmost districts of Sweden, younger than 73 years of age. As MC and auto-immunity are more frequent in women than in men [9], [14], and as the health-related quality of life and the experience of symptoms differ between the genders [15], we chose to include only women in the study.

### Patient Recruitment and Study Design

Between March and June 2011, invitations, including information on the study and questionnaires about social and life style factors, gastrointestinal symptoms, and medical condition and medication were sent by mail to all 240 women. A reminding letter was sent a month after the invitation letter to those who had not answered. They were also invited to provide blood samples for routine analyses according to standardised methods at the Division of Clinical Chemistry, Department of Laboratory Medicine in Skåne, at their local hospital. Analyses for IgG auto-antibodies were performed. ANA, ANCA, and ASCA were analysed at the Division of Immunology, Department of Laboratory Medicine in Skåne, Lund, and analyses of anti-IA2, anti-GAD and anti-TPO antibodies, and TRAK were performed at the Division of Clinical Chemistry, Department of Laboratory Medicine in Skåne, Malmö.

Medical records were scrutinised, and age, gastrointestinal symptoms, examinations, and treatments were recorded. Patients were divided into two groups. One group included patients with at least two episodes of watery diarrhoea; and/or those who were dependent on long-term treatment of corticosteroids to maintain remission; and/or those who had had two pathological intestinal mucosa biopsies (MC1, n = 73). The other group included patients who had concomitant coeliac disease [11], had undergone an acute gastroenteritis shortly before the diagnostic colonoscopy [4], had had only one episode of severe diarrhoea, or had had a normal biopsy after the initial pathological intestinal biopsy, and were in clinical remission (MC2, n = 60).

### Immunological Analyses

Healthy blood donors served as controls for all immunological analyses, except for TRAK, for which control values in healthy controls are available by the same method [16]. Cut-off levels and prevalence figures in the healthy population are the reference values at the Department of Laboratory Medicine in Skåne after in-house examinations. Our reference values for anti-GAD and anti-IA2 antibodies have been published previously [17].

ANA and ANCA were analysed by indirect immunofluorescence (IIF) [18], [19]. The cut-off value for ANA, >14 IU/ml, is set at the dilution where 5% of blood donors (50 men and 50 women) showed a positive ANA. The assay is calibrated against the international WHO reference for homogeneous ANA. Cytoplasmic (C)-and peri-nuclear (P)-ANCA have reference values of >10 IU/ml. The cut-off is determined by the supplier (Euro Immune AG, Lübeck, Germany) and of 50 healthy blood donors tested, none showed a positive result. ANA and ANCA were analysed in accordance with the International Organisation for Standardisation (ISO)/International Electrotechnical Commission (IEC) 17025 (General requirements for the competence for testing and calibration laboratories).

ASCA is analysed by an automated ELISA method for which the reference value is set to >10 IU/ml in accordance with the manufacturer’s recommendation (Orgentec Diagnostika, Alegria®, Mainz, Germany), and both IgA and IgG levels are given. Of 50 healthy blood donors tested at our laboratory, 8% were positive for both ASCA IgA and IgG, whereas other studies have found 0.6%–3.1% positive among healthy blood donors by the same method [20].

Antibodies against IA2 and GAD were analysed with a commercial ELISA kit (RSR Ltd, Cardiff, UK) according to the manufactureŕs manual for which the cut-off values are defined as >15 and >11 kIU/L, respectively [12]. In a cohort of 120 healthy Swedish women, median age 32 years, range 19–44 years, none was positive for anti-GAD antibodies and 31% were positive for anti-IA2 antibodies [17].

Anti-TPO antibodies are analysed by a chemiluminiscence enzyme immunological method (Roche Diagnostics Limited, cobas®, West Sussex, UK) and TRAK by an RIA method (Thermo B.R.A.H.M.S., TRAK Human RIA) [16]. Anti-TPO antibodies are found in 7% of blood donors when the cut-off level is set to >70 kIU/L [21]. The reference value for TRAK with a sensitivity of 98.8% is set to >1.5 IU/L, and <1% of 282 healthy individuals of median age 45 years, range 20–73 years, were positive [16].

### Questionnaires

A self-administered questionnaire about marital status, education, employment, smoking habits, wine consumption, physical activity, medical conditions and medication was completed by the patients. The patients were asked the questions: “ Have you ever been treated for any of the following diseases, namely, hypertension, allergy, thyroid disorders, fracture, rheumatoid arthritis, asthma, gastric ulcer, ovarial inflammation, herpes infection, endometriosis, Chlamydia Trachomatis, kidney stones, coeliac disease, diabetes mellitus type 1 or 2, myocardial infarction, intermittent claudication or stroke”. A list of the drugs, vitamins and minerals currently being taken was completed. The participants were also asked about heredity for cancer, stroke, myocardial infarction, hypertension, diabetes mellitus, thyroid disorders, asthma, rheumatoid arthritis, IBD or coeliac disease.

#### Gastrointestinal symptom rating scale (GSRS)

The GSRS is a Swedish, disease-specific and self-administered questionnaire, designed to evaluate perceived severity of gastrointestinal symptoms during the previous week [22], [23], [24]. The questionnaire includes 15 items and uses a 7-grade Likert scale. This gives a total range value between 15 and 105 where the highest score (seven) represents the most pronounced symptoms and the lowest (one) no symptoms. The items are divided into five dimensions representing Reflux Syndrome (two items), Abdominal Pain Syndrome (three items), Constipation Syndrome (three items), Indigestion Syndrome (four items) and Diarrhoea Syndrome (three items). The data are presented as the average of the total score divided by the number of items. Norm values for the healthy, gender-matched population are available [25].

#### Rome III criteria

The patients completed an abbreviated version of the Rome III questionnaire, including only irritable bowel syndrome (IBS) symptoms [26]. This questionnaire has been translated and validated into the Swedish language (Magnus Simrén and Anna Rydén). Patients who fulfilled the criteria in the Rome III questionnaire were classified as also suffering from IBS-like symptoms.

### Statistical Analyses

The data were analysed using the statistical software package SPSS for Windows^©^ (Release 20.0; IBM). Values are given as mean ± standard deviation (SD) or median (interquartile range) depending on distribution norm. Gastrointestinal symptoms are given as the mean with a 95% confidence interval, because the reference values in reference No 25 are given in this form. All patients who expressed any form of measured auto-antibody were classified as antibody positive, and calculations of the association between antibodies and clinical features were performed both for each antibody and for the total presence of antibodies. Differences between groups were calculated by the 2-tailed Mann-Whitney *U* -test or Student *t-* test. The Spearman correlation test was used to examine correlations between groups. Fisheŕs exact test was used to examine differences between the presence of auto-antibodies in diseases and heredity; differences in the prevalence of auto-antibodies and diseases between CC and LC; and differences in the prevalence of auto-antibodies between MC1 and MC2. Due to several hypotheses tested, p<0.01 was considered statistically significant.

## Results

### Patient Characteristics

In total, the 133 women with MC, who provided blood samples, were included in the study (median age 63 (59–67) years, range 27–73 years). CC was diagnosed in 77 patients (58%) (median age 64 (59–68) years, range 31–73 years) and LC in 56 patients (42%) (median age 63 (54–67) years, range 27–72 years). Of these 133, 69 patients (52%) also fulfilled the Rome III criteria for IBS, 40 of the patients (52%) with CC and 29 of the patients (52%) with LC. The duration of gastrointestinal symptoms at study inclusion was 8 (3–14) years. There was no correlation between duration of symptoms and age (correlation coefficient = −0.097, p = 0.286). The body mass index was 24.8 (22.7–28.7) kg/m^2^. Of the patients, 25% were regular smokers, 8% smoked occasionally, 40% had stopped smoking and 27% had never smoked. There was no difference in smoking habits between CC and LC (p = 0.350). Routine analyses were within the reference values used in the laboratory in the vast majority of cases (Table 1).

**Table 1:** Prevalence of auto-antibodies in microscopic colitis.

	Controls	Microscopiccolitis	Collagenouscolitis	Lymphocyticcolitis	P- value
		n = 133	n = 77	n = 56	
**Hb (**117–153****g/L)		132.2±9.3	132.2±9.5	132.2±9.0	0.987
**Leucocytes**					
(3.5–8.8×10^9^/L)		6.9±2.0	7.1±1.9	6.8±2.1	0.430
**S-Albumin**					
(36–48 g/L)		39.9±3.3	39.9±3.6	40.0±3.0	0.794
**CRP** (<3.1 mg/L)		2.0 (1.0–4.0)	4.0 (1.0–4.0)	1.6 (1.0–2.9)	0.014
**P-Sodium**		140.0	141.0	140.0	0.111
(137–145 mmol/L)		(139.0–42.0)	(139.0–142.0)	(139.0–142.0)	
**P-Potassium**					
(3.5–4.4 mmol/L)		3.9±0.4	3.9±0.3	3.9±0.4	0.916
**ANA**					
(≤14 IU/ml)(n, %)	(5)^α^	19 (14)	8 (10)	11 (20)	0.140
**C-ANCA**					
(≤10 IU/ml)(n, %)	(0)^β^	1 (1)	1 (1)	0 (0)	1.000
**P-ANCA**					
(≤10 IU/ml) (n, %)	(0)^β^	7 (5)	4 (5)	3 (5)	1.000
**ASCA IgA**					
(≤10 IU/ml) (n,%)	(8)^β^	0 (0)	0 (0)	0 (0)	
**ASCA IgG**					
(≤10 IU/ml)(n, %)	(8)^β^	17 (13)	7 (9)	10 (18)	0.187
**Anti-GAD**					
(≤11 kIU/L) (n, %)	(0)^γ^	7 (5)	4 (5)	3 (5)	1.000
**Anti-IA2**					
(≤15 kIU/L) (n, %)	(31)^γ^	2 (2)	1 (1)	1 (2)	0.100
**Anti-TPO**					
(≤70 kIU/L) (n, %)	(7)^β^	18 (14)	9 (12)	9 (16)	0.444
**TRAK**					
(≤1.5 IU/L)(n, %)	(1)^δ^	1 (1)	0 (0)	1 (2)	

ANA = anti-nuclear antibodies, C-ANCA = cytoplasmic anti-neutrophil cytoplasmic antibodies, ASCA = anti-Saccharomyces cerevisiae antibodies, CRP = C-reactive protein, anti-IA2 =  anti-islet antigens-like insulin 2, anti-GAD = anti-glutamic acid decarboxylase, Hb = haemoglobin, IgA = Immunglobulin A, IgG = Immunglobulin G, P = plasma, P-ANCA = peri-nuclear anti-neutrophil cytoplasmic antibodies, S = serum, anti-TPO = anti-thyroid peroxidase, TRAK = thyrotropin receptor antibodies. Reference values in brackets. n (%) = number (percent) of positive antibodies. Controls consisted of: α = healthy blood donors, 50 men and 50 women, β = 50 healthy blood donors, γ = 120 healthy women, reference No 17, δ = 282 healthy controls from reference No 16. Values are given as mean ± standard deviation or median (interquartile range). Student *t-* test, Mann-Whitney *U-* test or Fisheŕs exact test were used for comparisons between collagenous colitis and lymphocytic colitis. P<0.01 was considered statistically significant.

### Prevalence of Auto-antibodies

The highest prevalence of antibodies was found of ANA, ASCA IgG, and anti-TPO antibodies, where the prevalence was doubled in the patients compared to the general population, with no differences between CC and LC, or between MC1 and MC2 (Table 1 and Table 2). There was no difference between CC and LC in the total presence of auto-antibodies (p = 0.375). The median age in ANA-positive patients tended to be higher than in ANA-negative patients (67 (62–71) years and 63 (56–67) years, respectively, p = 0.013). In contrast, the median age tended to be lower in ASCA-positive patients compared to ASCA-negative patients (59 (46–66) years and 64 (59–68) years, respectively, p = 0.031). The relatively high prevalence of anti-TPO antibodies was not reflected by such a difference in age in patients who expressed antibodies compared to those who did not (60 (47–66) years and 64 (59–68) years, respectively, p = 0.097).

**Table 2:** Prevalence of auto-antibodies in persistent (MC1) and transient microscopic colitis (MC2).

	MC1	MC2	P-value
	n = 73	n = 60	
**Age** (years)	63 (57–67)	64 (59–68)	0.405
**Hb (**117–153****g/L)	132.3±8.8	132.1±9.9	0.916
**Leucocytes**			
(3.5–8.8×10^9^/L)	7.0±2.3	6.9±1.5	0.906
**S-Albumin** (36−48g/L)	40.1±3.5	39.7±3.1	0.494
**CRP** (<3.1 mg/L)	2.0 (1.0−4.0)	2.0 (1.0−3.5)	0.840
**P-Sodium**			
(137−145 mmol/L)	141.0 (139.0−142.0)	140.0 (139.0−142.0)	0.253
**P-Potassium**			
(3.5−4.4 mmol/L)	3.9±0.3	3.9±0.4	0.736
**ANA** (≤14 IU/ml)(n, %)	12 (16.4)	7 (11.7)	0.619
**C-ANCA**			
(≤10 IU/ml)(n, %)	1 (1.4)	0 (0)	1.000
**P-ANCA**			
(≤10 IU/ml)(n, %)	5 (6.8)	2 (3.3)	0.463
**ASCA IgA**			
(≤10 IU/mL)(n, %)	0 (0)	2 (3.3)	
**ASCA IgG**			
(≤10 IU/mL)(n, %)	11 (15.1)	6 (10)	0.602
**Anti-GAD**			
(≤11 kIU/L)(n, %)	5 (6.8)	2 (3.3)	0.460
**Anti-IA2**			
(≤15 kIU/L)(n, %)	0 (0)	2 (3.3)	0.100
**Anti-TPO**			
(≤70 kIU/L)(n, %)	9 (12.3)	9 (15.0)	0.799
**TRAK** (≤1.5 IU/L)(n, %)	0 (0)	1 (1.7)	0.456

ANA = anti-nuclear antibodies, C-ANCA = cytoplasmic anti-neutrophil cytoplasmic antibodies, ASCA = anti-Saccharomyces cerevisiae antibodies, CRP = C-reactive protein, anti-IA2 =  anti-islet antigens-like insulin 2, anti-GAD = anti-glutamic acid decarboxylase, Hb = haemoglobin, IgA = Immunglobulin A, IgG = Immunglobulin G, P = plasma, P-ANCA = peri-nuclear anti-neutrophil cytoplasmic antibodies, S = serum, anti-TPO = anti-thyroid peroxidase, TRAK = thyrotropin receptor antibodies. Reference values in brackets. n (%) = number (percent) of patients with positive antibodies. Values are given as mean ± standard deviation or median (interquartile range). Student *t-* test, Mann-Whitney *U-* test or Fisheŕs exact test. P<0.01 was considered statistically significant.

When examining the most prevalent concomitant diseases, excluding sporadic diseases, ANA-positive patients also suffered from hypertension (nine), allergy (seven), thyroid disorders (six), rheumatoid arthritis (three), and coeliac disease (three). Patients expressing ASCA IgG also suffered from hypertension (eight), rheumatoid arthritis (four), asthma (three), coeliac disease (three), and allergy (three). Anti-TPO antibodies were found together with thyroid disorders in seven patients, with allergy in four and with asthma and rheumatoid arthritis in three. Of the seven who expressed P-ANCA, four suffered from allergy, and three from thyroid disorders, rheumatoid arthritis, and hypertension, respectively. Of the seven patients who expressed anti-GAD antibodies, five also suffered from diabetes mellitus. One of the patients with anti-IA2 antibodies also expressed anti-GAD antibodies, and suffered from diabetes mellitus. Only eight of the patients with any antibody had MC as a sole disease. The patient with the most concomitant diseases suffered from seven of these, apart from MC and IBS. There was no difference in the results of routine blood analyses between patients with and those without auto-antibodies (Table 3).

**Table 3:** Laboratory values and gastrointestinal symptoms in relation to the presence of auto-antibodies.

	Antibodies	No antibodies	P-value
	n = 56	n = 77	
**Age** (year)	62 (58−68)	63 (58−67)	0.845
**Hb (**117−153****g/L)	132.5±9.3	132.0±9.1	0.764
**Leucocytes**			
(3.5−8.8×10^9^/L)	6.9±2.1	7.0±1.9	0.791
**S-Albumin**			
(36−48 g/L)	40.0±3.3	40.0±3.4	0.966
**CRP** (<3.1 mg/L)	2.0 (1.0−4.4)	2.0 (1.0−3.5)	0.056
**P-Sodium**			
(137−145 mmol/L)	141.0 (139.0−143.0)	140.0 (139.0−142.0)	0.253
**P-Potassium**			
(3.5−4.4 mmol/L)	3.9±0.3	3.9±0.4	0.907
**Duration** (years)	10.0 (5.8−16.0)	6.0 (3.0−12.0)	0.038
**Abdominal pain**	2.55 2.28−2.83	2.63 2.37−2.88	0.428
**Indigestion**	3.01 2.76−3.26	2.92 2.66−3.18	0.372
**Reflux**	2.50 2.17−2.83	2.30 2.04−2.56	0.195
**Constipation**	3.33 2.92−3.74	3.40 3.04−3.76	0.457
**Diarrhoea**	4.02 3.68−4.35	3.90 3.55−4.24	0.372
**GSRS Total**	8.92 8.04−9.80	9.30 8.69−9.92	0.299

Values are given as median (interquartile range) or mean with a 95% confidence interval. GSRS = Gatrointestinal Symptom Rating Scale. Mann-Whitney *U*-test or Student *t* -test. P<0.01 was considered statistically significant.

### Prevalence of Other Diseases

The distribution of present and past diseases is shown in Table 4. More than half of the patients fulfilled the criteria both for MC and IBS. Apart from IBS, only 26 of the patients (20%) reported that they had had MC as the sole disease. No difference in the prevalence of different diseases was found between the CC and LC groups (Table 4). Three of the 10 patients with diabetes mellitus had auto-immune type 1 diabetes, and the rest had type 2 diabetes. There was no association between the total presence of antibodies and any of the diseases investigated in the statistical calculations (Table 4), or between each antibody and disease (data not shown). The patients were, at the time of the study, treated with many drugs simultaneously, where corticosteroids (30%), proton pump inhibitors (22%), angiotensin-converting enzyme inhibitors or angiotensin II receptor antagonists (19%), thyroid hormones (19%), anti-depressant drugs of the type selective serotonin reuptake inhibitors (17%), statins (17%), and beta-blockers (14%) were the most frequently used. Treatment with corticosteroids tended to be more prevalent in MC1 (26) than in MC2 (11) (p = 0.033).

**Table 4:** The prevalence of different past and current diseases in microscopic colitis and its association with auto-antibodies (ab).

	Collagenous	Lymphocytic	P-value	Microscopic	P-value*
	colitis (CC)	colitis (LC)	CC vs	colitis (MC)	ab vs MC
	n = 77	n = 56	LC	n = 133	
**Age** (year)	64 (59−68)	63 (54−67)	0.080	63 (59−67)	0.845
**IBS**	40 (52)	29 (52)	1.000	69 (52)	0.482
**Hypertension**	27 (35)	14 (25)	0.173	41 (31)	0.033
**Allergy**	29 (38)	12 (21)	0.075	41 (31)	1.000
**Thyroid**	14 (18)	16 (29)	0.208	30 (23)	0.085
**disorder**					
**Fracture**	13 (17)	14 (25)	0.372	27 (20)	0.176
**Rheumatoid**	13 (17)	13 (23)	0.500	26 (20)	0.258
**arthritis**					
**Asthma**	15 (19)	7 (12)	0.343	22 (16)	0.810
**Gastric ulcer**	11 (14)	10 (18)	0.808	21 (16)	0.327
**Ovarial**	13 (17)	6 (11)	0.321	19 (14)	0.323
**inflammation**					
**Herpes**	6 (8)	13 (23)	0.022	19 (14)	0.330
**infection**					
**Endometriosis**	8 (10)	5 (9)	1.000	13 (10)	0.235
**Chlamydia**	6 (8)	6 (11)	0.763	12 (9)	0.762
**Kidney stones**	9 (12)	2 (4)	0.116	11 (8)	0.346
**Cancer**	8 (10)	3 (5)	0.347	11 (8)	0.115
**Coeliac disease**	2 (3)	8 (14)	0.017	10 (8)	0.314
**Diabetes**	5 (6)	5 (9)	1.000	10 (8)	0.514
**mellitus**					
**Type 1**	2 (3)	1 (2)		3 (2)	
**Type 2**	3 (4)	4 (7)		7 (5)	
**Myocardial**	6 (8)	2 (4)	0.464	8 (6)	1.000
**infarction**					
**Intermittent**	3 (4)	3 (5)	1.000	6 (4)	0.222
**claudication**					
**Stroke**	3 (4)	2 (4)	1.000	5 (4)	0.646

n (%) = number (percent) of patients with the disease.

IBS = Irritable bowel syndrome,

* = Comparison of prevalence of diseases in the whole MC group between patients with or without any auto-antibodies. Mann-Whitney *U*-test and Fisheŕs exact test. P<0.01 was considered statistically significant.

There was no difference in heredity concerning cancer (60%) (p = 0.648), hypertension (61%) (p = 0.208), myocardial infarction (50%) (p = 0.031), stroke (36%) (p = 0.627), diabetes mellitus (32%) (p = 0.217), rheumatoid arthritis (34%) (p = 0.665), thyroid disorders (31%) (p = 0.870), asthma (29%) (p = 0.676), IBD (28%) (p = 0.260) or coeliac disease (10%) (p = 1.000) between patients with and those without antibodies.

### Prevalence of Gastrointestinal Symptoms

Patients with MC had more gastrointestinal symptoms than healthy females in all the dimensions Reflux Syndrome, Abdominal Pain Syndrome, Constipation Syndrome, Indigestion Syndrome, and Diarrhoea Syndrome (Table 5). There was no difference in symptoms between CC and LC (Table 5). There was no difference in disease duration and degree of gastrointestinal symptoms between patients with and those without any kind of auto-antibodies (Table 3).

**Table 5:** Values of the Gastrointestinal Symptom Rating Scale (GSRS) in patients with microscopic colitis and healthy females.

	Microscopic	Collagenous	Lymphocytic	Healthy fem
	colitis	colitis	colitis	
	Mean 95% CI	Mean 95% CI	Mean 95% CI	Mean 95% CI
**Abdominal pain**	2.60	2.53	2.70	1.63
	2.42−2.79	2.30−2.77	2.39−3.00	1.58−1.68
**Indigestion**	2.96	3.00	2.90	1.78
	2.78−3.14	2.76−3.24	2.63−3.18	1.73−1.83
**Reflux**	2.40	2.37	2.45	1.37
	2.20−2.60	2.11−2.62	2.11−2.78	1.33−1.42
**Constipation**	3.37	3.13	3.70	1.65
	3.11−3.64	2.80−3.46	3.27−4.13	1.59−1.71
**Diarrhoea**	3.94	4.00	3.87	1.39
	3.70−4.18	3.67−4.33	3.50−4.23	1.35−1.43
**GSRS Total**	9.16	8.91	9.49	1.56
	8.65−9.67	8.21−9.61	8.75−10.23	1.52−1.59

CI = Confidence interval. Data for healthy females referred to in reference No 25.

## Discussion

The main finding of this cross-sectional study was that the prevalence of common auto-antibodies such as ANA, P-ANCA, ASCA IgG, and anti-GAD and anti-TPO antibodies is slightly more prevalent in MC patients than in the general Swedish population, but they are nonetheless present in only a minority of cases. C-ANCA, ASCA IgA, anti-IA2 antibodies, and TRAK are only occasionally found. There was no difference in gastrointestinal symptoms between patients with and those without auto-antibodies. The antibodies were present in patients who also suffered from other concomitant diseases.

Auto-immunity has been suggested as a plausible aetiology of MC, as an association with the same HLA genotypes in MC as described in other known auto-immune diseases has been described [2], [9]. However, the prevalence of C-ANCA, ASCA IgA, anti-IA2 antibodies, and TRAK was in the same range or lower as in the general population, according to our laboratory reference values of healthy blood donors, as has also been described in previous studies [3], [7], [11], [13], [16], [17]. Patients predisposed to auto-immunity usually express higher levels of several antibodies in serum [5], [6], [7], [8]. The prevalence of ANA, P-ANCA, ASCA IgG, and anti-GAD and anti-TPO antibodies were slightly increased compared to healthy controls, but not at all to the same magnitude as in patients with Crohńs disease, ulcerative colitis, diabetes mellitus or thyroid disorders [7], [8], [12], [27], [28]. Furthermore, reference values are set in relation to the general population, including both genders and all ages. In this study of women of upper middle age, the prevalence of auto-antibodies is higher than in the general population [14]. World-wide, 12%–26% of healthy women and 3%–14% of healthy men have anti-TPO antibodies [28], and 6% of healthy women are considered positive for ANA [29]. The increased prevalence of antibodies in our present study can be explained by other concomitant diseases of auto-immune character, e.g. rheumatoid arthritis, coeliac disease, thyroid disorders and diabetes mellitus, and by our cohort being of upper middle-aged women. As our older MC patients had many concomitant diseases, not only of auto-immune origin, it is difficult to estimate the true association with auto-immune diseases. The associations observed could be due to secondary effects on the digestive tract of the diseases, or their treatment, and do not necessarily mean a common causal aetiology [1]. Smoking *per se* is associated with a higher prevalence of ANA [30], and this could explain the higher ANA positivity in our study with a high frequency of former and current smokers. Thus, our finding in the present study does not indicate that auto-immunity is the most important cause of MC.

Previously described associations between MC and drug treatment and smoking [31], [32], and the higher age in the diseased patients [9], suggest environmental and circulatory influences as important aetiological factors for MC [33]. Intestinal ischemia should also be considered, especially as smoking is a risk factor for the disease [32], and the patients are older, with a high prevalence of cardiovascular diseases [33]. The high prevalence of using angiotensin-converting enzyme inhibitors or angiotensin II receptor antagonists and beta-blockers in the current study, may also affect intestinal blood circulation, with secondary tissue effects.

Several conditions, including surgical manipulation of the tissue, *Helicobacter pylori*, infections and drugs may lead to intestinal lymphocytosis [1], [34]. In clinical practice, patients with a histopathological diagnosis of MC are clinically classified as MC, irrespective of whether the lymphocytosis is primary or secondary. Furthermore, 63% of patients have had only a single attack of MC [35]. In other prospective studies, there were high relapse rates after completion of treatment with budesonide [36], [37], and some patients have refractory and severe symptoms requiring surgery [9]. This must indicate the necessity to restrict our future classification of MC. The diagnosis of MC should probably include only true conditions of consistent, permanent intestinal lymphocytosis, without any other plausible explanations [1], [33], [38]. However, we did not find any differences between MC1 and MC2 in antibody prevalence.

In this study, the patients represent the vast majority of female MC patients younger than 73 years in Skåne, independent of whether they are treated at primary care or tertiary centres. This may explain the lower prevalence of auto-immune diseases and antibody prevalence compared to other studies representing tertiary centres, in which studies of only auto-immune concomitant disorders are reported [3], [4], [7], [39]. Previous studies, in which a higher prevalence of coeliac disease has been described, patients were younger and were screened by endoscopic biopsies specifically to detect coeliac disease. Coeliac disease is characterised by villous atrophy in the small intestine, and the colonic lymphocytosis may reflect the same reaction of the large intestine, rather than a distinct disease [1], [39]. Our patient cohort has a high prevalence of IBS-like symptoms, and among these patients there may be hidden some cases of un-investigated coeliac disease, not diagnosed by serology. As IBS is most often found in younger persons and is characterised by no organic findings [26], we have chosen to call the presence of abdominal pain and bloating IBS-like symptoms. These symptoms are non-specific, and may depend on side effects of drug treatment rather than a different and distinct disease.

The peak incidence of many of the concomitant diseases is at a younger age, whereas the peak incidence of MC is at 65 years [9], [14], [40]. One limitation of this and most other studies in this field is the retrospective or cross-sectional character, making it difficult to know which disease, or treatment, debuted first. Furthermore, if the patient is not referred to colonoscopy, the diagnosis IBS is set instead of MC. In many cases, the patients have had gastrointestinal symptoms for several years prior to colonoscopy, making the peak age of incidence falsely higher. Patients with concomitant diseases, already treated for these at a hospital, are probably referred for a colonoscopy sooner after debut of another symptom than an otherwise healthy individual, and thus the diagnosis is most often set in these cases.

The small differences between CC and LC in this study concerning concomitant diseases and serological markers, and in our previous study concerning gastrointestinal symptoms and psychological well-being [41], suggest that the two forms of the disease are not markedly heterogenous. A transition between the subtypes over time has been described, and that perhaps the two forms should be considered as one entity aetiologically [38]. The classification as CC and LC may reflect a histopathologic classification rather than a clinically relevant distinction [33].

In conclusion, although they showed a slightly increased prevalence of some auto-antibodies, the majority of our MC patients did not express any of the antibodies measured. The higher prevalence of auto-antibodies measured may be explained by other, concomitant, auto-immune diseases, a high frequency of smokers, and the composition of the cohort being of women of late middle age. Future research in this field should focus on prospective studies in that fraction of patients with MC only, to find out whether auto-immunity is the aetiology in these cases.
